# Time series non-Gaussian Bayesian bivariate model applied to data on HMPV and RSV: a case of Dadaab in Kenya

**DOI:** 10.1186/s12889-019-7036-2

**Published:** 2019-06-24

**Authors:** Raymond Nyoka, Thomas N. O. Achia, Jimmy Omony, Samuel M. Musili, Anthony Gichangi, Henry Mwambi

**Affiliations:** 1 School of Mathematics, Statistics and Computer Science, University of KwaZulu-Natal, Private Bag X01, Scottsville, 3209 South Africa; 2 Molecular Genetics Department, University of Groningen, 9747 AG Groningen, The Netherlands; 3 Statistics Department, Jomo Kenyatta University of Agriculture and Technology, P.O. Box 62000-00200, Nairobi, Kenya; 4 Jhpiego - an affiliate of John Hopkins University, P.O. Box 66119, Westlands, Nairobi, 00800 Kenya; 5 Nairobi, Kenya

**Keywords:** Non-Gaussian bivariate Bayesian model, RSV, HMPV, Epidemic, Time series, Climatic factors

## Abstract

**Background:**

Human metapneumovirus (HMPV) have similar symptoms to those caused by the respiratory syncytial virus (RSV). The modes of transmission and dynamics of time series data still remain poorly understood. Climatic factors have long been suspected to be implicated in impacting on the number of cases for these epidemics. Currently, only a few models satisfactorily capture the dynamics of time series data of these two viruses. Our objective was to assess the presence of influence of high incidences between the viruses and to ascertain whether higher incidences of one virus are influenced by the other.

**Methods:**

In this study, we used a negative binomial model to investigate the relationship between RSV and HMPV while adjusting for climatic factors. We specifically aimed at establishing the heterogeneity in the autoregressive effect to account for the influence between these viruses.

**Results:**

In this study, our findings showed that RSV incidence contributed to the severity of HMPV incidence. This was achieved through comparison of 12 models with different structures, including those with and without interaction between climatic factors. The models with climatic factors out-performed those without.

**Conclusions:**

The study has improved our understanding of the dynamics of RSV and HMPV in relation to climatic cofactors thereby setting a platform to devise better intervention measures to combat the epidemics. We conclude that preventing and controlling RSV infection subsequently reduces the incidence of HMPV.

## Background

Epidemiological knowledge of the respiratory system has been mostly related to developed countries, though the burden of respiratory virus infections (RVIs) is more manifested in developing countries with very high hospitalization and mortality rates [1]. Higher mortality is associated with increased displacement into overcrowded refugee camps [2]. The burden of RVIs is considerably high during crises times [3] and is more severe in infants [4]. Recently, Pastula et al. [5] highlighted that hospitalization for respiratory syncytial virus (RSV) is not limited to infants but also includes adults. In 2001, HMPV was identified as a potential etiologic agent for respiratory infections [6]. A study at Queen Mary Hospital in Hong Kong showed that the peaks of HMPV and that of RSV activity occurred in spring and the early months of summer and viral diagnoses during the study period showed that RSV and HMPV had similar seasonality [7]. Guerrero et al. [8] indicate that RSV but not HMPV induces a productive infection in human monocyte-derived dendritic cells. Reinfection by RSV has a great impact on human health and may cause long-term effects on the host immune response [9]. Greensill et al. [10] detected HMPV in 21 out of 30 infants infected with severe RSV and were hospitalized requiring intensive-care unit ventilator support. Konig et al. [11] found out that 60% of the cases with HMPV had RSV. They also found that HMPV contributed to the severity of Lower respiratory tract infections (LRTIs) at a lower rate than RSV and coinfection was considered a cause of severe lower respiratory tract disease. The HMPV infections have similar symptoms to those caused by RSV [12, 13], they share similar risk factors [14] and simultaneous detection times [15]. The HMPV and RSV may cross-react directly or indirectly because they are both co-viruses to each other [16]. The correlation between RSV and HMPV in the refugee settings and even in the tropical region has not been studied. We specifically aimed at establishing the heterogeneity in the autoregressive effect to account for the influence between these viruses. The modelling of the time series events of these viruses will not only help in the prediction of their outbreaks but also in estimating which outbreaks precede each other. The results could be used by other countries in the tropical zone of Africa with similar settings to inform control measures to prevent outbreaks.

In Section 2, we show the data source and the statistical model fitting with and without climatic covariates to a bivariate time series. In Section 3, we show the applicability of the models illustrated with a real-world example and the results obtained. In Section 4, we discuss the results and finally conclude in Section 5.

## Methods

### Data

A surveillance system for viral respiratory illnesses that included RSV and HMPV was implemented in a refugee camp in Dadaab located in northeastern province of Kenya from September 2007 to August 2011. Both paediatric and adult patients presenting to a medical unit and who met the case definition for influenza-like illness or severe acute respiratory infection were enrolled in the surveillance. Laboratory confirmed test results for RSV and HMPV were obtained after adults and guardians of all minors filled a consent form. The number of laboratory-confirmed cases was recorded every day. In this analysis, only the monthly counts of RSV and HMPV cases among children younger than 5 years were considered. Local weather and climatic data from a neighboring weather station were obtained from the World Meteorological Organization’s (WMO’s), World Weather Watch Program, according to WMO Resolution 40 (Cg-XII) (available at http://www7.ncdc.noaa.gov/CDO/cdo). The meteorological dataset was recorded on a daily basis and aggregated monthly for the purpose of this analysis. The variables included the mean temperature, mean dew point for the day (both in ^0^F), the mean sea level pressure for the day in millibars, the mean visibility for the day in miles, the mean wind speed for the day in knots, the minimum and maximum temperature (°F) reported during the day and the total precipitation (in inches).

### Statistical modeling

In this paper, we used surveillance data aggregated by month in a time series model and the negative binomial distribution to address the issue of over-dispersion. We model the relationship between the two viruses namely, RSV and HMPV. Meteorological variables were included in the model to help assess for serial correlation. Held et al. [17] suggested that environmental factors can be incorporated into these models to improve model fit to data and predictions. These models help to assess the presence of influence of high incidences between the viruses and whether higher incidences of one virus are influenced by another. They also aid in evaluating if an epidemic component can be isolated within or between the viruses and how the autoregressive component captures the residual temporal dependence in the time series after adjusting for seasonal effects. Modeling count data is faced with many challenges since count outcomes do not meet the usual normality assumption required of many standard statistical tests. Typical log-transformation to induce normality does not often work or categorization of the outcome may lead to loss of information as described by O’Hara and Kotze [18]. The most commonly used models to study the dynamics of epidemics and predict future outbreaks using count data are the Poisson [19] and the negative binomial distributions [20]. We modelled the time-evolution of two epidemics using a bivariate approach suggested by Held et al. [17]. We assume that we have *i* = 1, … , *m* ‘viruses’ and denote with *y*_*it*_ the number of cases in virus *i* at time *t*. The general model for the multiple time series of count events {*y*_*it*_, *i* = 1,  … , *m*; *t* = 1,  … , *T*} for virus type *i* at time *t* assumes a Poisson distribution with conditional mean *μ*_*it*_ given by

1 $$ \log \left(\ {\mu}_{it}\right)={\lambda}_{i,t-1}{y}_{i,t-1}+{\phi}_{i,t-1}\sum \limits_{j\ne i}{\omega}_{ij}{y}_{j,t-1}+{\eta}_{i,t}{\nu}_{it}. $$

It holds VAR(*y*_*i*, *t*_|*y*_*i*, *t* − 1_) = E(*y*_*i*, *t*_| *y*_*i*, *t* − 1_) = *μ*_*it*_. Hence, in the case of a conditional Poisson response model the conditional mean *μ*_*it*_, is identical to the conditional variance *δ* of the observed process.

In model 1, *λ*_*i*, *t* − 1_ is the autoregressive parameter representing the proportion of epidemic cases from the total number of cases for virus type *i* at time *t.* When *λ*_*i*, *t* − 1_ ≥ 1 (an outbreak occurs) there is an influx of the endemic cases and *λ*_*i*, *t* − 1_ < 1 means the process is stable (no outbreak occurs). The *ϕ*_*i*, *t* − 1_ quantifies the influence of virus type *j* on *i*; *η*_*i*, *t*_ represents the monthly varying population counts of virus type *i* at time *t* (treated as an offset term in the model) and *ν*_*it*_ is the endemic component that explains the baseline incidence rate of cases as subsequently shown in eq. (5). The variable *y*_*j*, *t* − 1_ denotes the number of cases observed in virus type *j* at time *t* − 1. *ω*_*ij*_ is the weighting indicator and is equal to 1 if pathogens j and i have an autoregressive effect on each other and 0 otherwise.

This model is aggregation consistent where the aggregated counts ${y}_t=\sum \limits_{i=1}^m{y}_{it}$ have the mean,

$$ \log \left({\mu}_t\right)={\lambda y}_{t-1}+{\phi}_{t-1}{Z}_{t-1}+{\eta}_t{\nu}_t, $$

where, ${\boldsymbol{Z}}_{t-1}=\sum \limits_{j\ne i}{\omega}_{ij}{y}_{j,t-1},{\boldsymbol{\eta}}_t=\sum \limits_{i=1}^m{\eta}_{i,t},{\boldsymbol{\phi}}_t=\sum \limits_{i=1}^m{\phi}_{i,t},{\boldsymbol{\nu}}_t=\sum \limits_{i=1}^m{\nu}_{i,t}$. So, the parameter ***λ*** has the same interpretation for the aggregated counts similar to the counts *y*_*it*_.

In the presence of over-dispersion, the Poisson model is replaced by a negative binomial model where the conditional mean remains unchanged but the variance *δ* is modified to ***μ***_*t*_(1 + ***μ***_*t*_***ψ***) with over-dispersion parameter ***ψ*** > 0. The extent of over-dispersion is captured by how far the term ***ψ*** deviates from zero. An extensive discussion on handling over-dispersion can be found in the work of Ver Hoef and Boveng [21]. We are interested in two different types of viruses transmitted through the same route, i.e. respiratory illness. Let *x*_*k*, *t* − 1_ denote climatic covariates with ***τ***_*k*_ coefficients in the model and *k* = 1, … , *K* covariates. In the model, it is assumed that the cases follow a negative binomial distribution, ***y***_*t*_ ∣ ***y***_*t* − 1_~NegBin(***μ***_*t*_, ***ψ***), with conditional mean

2 $$ \mathit{\log}\left({\boldsymbol{\mu}}_t\right)={\boldsymbol{\lambda}}_{t-1}{\boldsymbol{y}}_{t-1}+{\boldsymbol{\tau}}_k{x}_{k,t-1}+{\phi}_{t-1}{\boldsymbol{Z}}_{t-1}+\mathit{\exp}\left({\boldsymbol{\eta}}_t\right) $$

and conditional variance

3 $$ {\boldsymbol{\mu}}_t\left(1+{\boldsymbol{\mu}}_t\boldsymbol{\psi} \right). $$

The incidence of the disease ***μ***_*t*_ was additively decomposed into two parts. The first part,

4 $$ {\xi}_t={\lambda}_{t-1}{y}_{t-1}+{\tau}_k{x}_{k,t-1}+{\phi}_{t-1}{z}_{t-1}, $$

is the epidemic component explaining the outbreaks or irregularities in the data including the interaction between the viruses. The second part is *ν*_*i*, *t*_ which is expressed in log-scale as

5 $$ \log \left({\nu}_{it}\right)={\alpha}_i+\sum \limits_{s=1}^S\left\{{\boldsymbol{\gamma}}_s\sin \left({\omega}_st\right)+{\boldsymbol{\delta}}_s\cos \left({\omega}_st\right)\right\}. $$

The endemic and epidemic components of the time series were explored and studied allowing for the separation of the regular pattern from irregular ones in estimating the epidemic peaks. The parameter *α*_*i*_ allows for different incidence levels of the viruses and *S* is the virus specific number of harmonic waves. The term in curly brackets captures seasonal variations. The ***γ***_*s*_ and ***δ***_*s*_ are the seasonal parameters while *ω*_*s*_ = 2*πs*/12 for monthly data are the Fourier frequencies.

### Likelihood and posterior distribution

The counts ***y***_*t*_, conditional on the previous observation ***y***_*t* − 1_ (Only lag one was applied in our case because more than one lag did not fit the data well) are assumed to follow a Negative binomial distribution with mean

6 $$ {\boldsymbol{\mu}}_t\boldsymbol{\theta} \equiv {\boldsymbol{\mu}}_t=\boldsymbol{\xi} +\boldsymbol{\nu}, \kern0.5em $$

where ***θ*** **=** (*θ*_1_,  … , *θ*_*m*,_*ψ*_1_,  … , *ψ*_*m*_)^*T*^ The log-likelihood of the observation ***y***_*t*_ is given as

7 $$ l\left(\boldsymbol{\theta} \right)=\sum \limits_t{l}_t\left(\boldsymbol{\theta}, \boldsymbol{\psi} \right) $$

and the likelihood as,

8 $$ f\left({\boldsymbol{y}}_t|\boldsymbol{\theta} \right)=\mathit{\exp}\left\{\sum \limits_t{l}_t\left(\boldsymbol{\theta}, \boldsymbol{\psi} \right)\right\}, $$

where,

9 $$ {l}_t\left(\boldsymbol{\theta}, \boldsymbol{\psi} \right)\propto \mathit{\log}\ \Gamma \left({\boldsymbol{y}}_t+\frac{1}{\boldsymbol{\psi}}\right)-\mathit{\log}\Gamma \left(\frac{1}{\boldsymbol{\psi}}\right)+\frac{1}{\boldsymbol{\psi}}\mathit{\log}\left(\frac{1}{1+{\boldsymbol{\psi} \boldsymbol{\mu}}_t\left(\boldsymbol{\theta} \right)}\right)+{\boldsymbol{y}}_t\mathit{\log}\left(\frac{{\boldsymbol{\psi} \boldsymbol{\mu}}_t\left(\boldsymbol{\theta} \right)}{1+{\boldsymbol{\psi} \boldsymbol{\mu}}_t\left(\boldsymbol{\theta} \right)}\right), $$

and Γ(.) is the gamma function and ***ψ*** and ***τ*** are the dispersion parameters. The gamma priors are assumed for ***ψ*** and ***τ***,

$$ \boldsymbol{\psi} \sim Ga\left({\alpha}_{\boldsymbol{\psi}, }{\beta}_{\boldsymbol{\psi}}\right), $$

$$ \boldsymbol{\tau} \sim Ga\left({\alpha}_{\boldsymbol{\tau}, }{\beta}_{\boldsymbol{\tau}}\right). $$

The virus dependent effects *α*_*i*_ are assumed to be independent and normally distributed with a large variance,

$$ \upalpha =\left({\upalpha}_1,\dots, {\upalpha}_{\mathrm{I}}\right)\sim \mathrm{N}\left(0,{\sigma}_{\upalpha}^2\mathrm{I}\right),{\sigma}_{\alpha}^2={10}^6, $$

where I is an identity matrix. All model parameters are non-negative and therefore we propose gamma prior distributions for them. The rate parameters ***λ***_*t*_ assumes independent gamma priors with gamma hyperpriors on the second parameter,

$$ {\boldsymbol{\lambda}}_t\sim Ga\left({\alpha}_{\boldsymbol{\lambda}, }{\beta}_{\boldsymbol{\lambda}}\right)\kern0.5em \mathrm{and}\kern0.5em {\beta}_{\boldsymbol{\lambda}}\sim Ga\left(a,b\right). $$

Where we use *α*_***λ***_ = 1, *a* = 10 and *b* = 10, with values for *α*_***λ***_, *a* and *b* chosen arbitrarily.

Independent normal priors are assumed for ***γ*** and ***δ***,

$$ \boldsymbol{\gamma} =\left({\gamma}_1,\dots, {\gamma}_I\right)\sim \mathrm{N}\left(0,{\sigma}_{\gamma}^2\mathrm{I}\right),{\sigma}_{\gamma}^2={10}^6, $$

$$ \boldsymbol{\delta} =\left({\delta}_1,\dots, {\delta}_I\right)\sim \mathrm{N}\left(0,{\sigma}_{\delta}^2\mathrm{I}\right),{\sigma}_{\delta}^2={10}^6. $$

The parameter ***ϕ***_*t*_ assumes gamma priors, ***ϕ***_*t*_~*Ga*(*α*_***ϕ***,_*β*_***ϕ***_).

The posterior distribution is therefore given as,

$$ f\left(\boldsymbol{\theta} |{\boldsymbol{y}}_t\right)\propto f\left({\boldsymbol{y}}_t|\boldsymbol{\theta} \right)f\left(\boldsymbol{\theta} \right), $$

which can be expressed as,

10 $$ {\displaystyle \begin{array}{l}f\left(\boldsymbol{\theta} |{\boldsymbol{y}}_t\right)\propto \mathit{\exp}\left\{\sum \limits_t{l}_t\left(\boldsymbol{\theta}, \boldsymbol{\psi} \right)\right\}\times \prod \limits_{s=1}^S{e}^{-\frac{1}{2c}{\sigma}_{\boldsymbol{\gamma}}^2}\times \prod \limits_{s=1}^S{e}^{-\frac{1}{2c}{\sigma}_{\boldsymbol{\delta}}^2}\times \prod \limits_{i=1}^m{e}^{-\frac{1}{2c}{\sigma}_{\alpha_i}^2}\\ {}\times \prod \limits_{i=1}^m{\lambda_i}^{\alpha_{\lambda_i}-1}{e}^{-{\beta}_{\lambda_i}^{\lambda_i}}{\lambda_i}^{a-1}{e}^{-b{\lambda}_i}\times \prod \limits_{i=1}^m{\psi_i}^{\alpha_{\psi_i}-1}{e}^{-{\beta}_{\psi_i}^{\psi_i}}\times \prod \limits_{i=1}^m{\phi_i}^{\alpha_{\phi_i}-1}{e}^{-{\beta}_{\phi_i}^{\phi_i}}\\ {}\times \prod \limits_{i=1}^m{\tau_i}^{\alpha_{\tau_i}-1}{e}^{-{\beta}_{\tau_i}^{\tau_i}}.\end{array}} $$

### Simulations

We investigated the proposed model performance on simulated data. We simulated bivariate data using a frequentist approach in R software using the package “Surveillance” previously used by Held et al. [22, 23]. We used the function “hhh4” with the class “disprog” to simulate two disease pathogen counts replicated 10,000 times. We then applied the Bayesian approach to compare different models based on varied scenarios. We considered a situation where there is the presence of overdispersion with the parameter *ψ*_*i*_ ≠ 0 assuming the negative binomial distribution and where *ψ*_*i*_ = 0 assumes the Poisson distribution. We also considered the presence and absence of the parameter *λ*_*i*_ (the ‘epidemic’ component) to evaluate temporal dependence. In this simulation, we disregarded the linear trend. It is evident from Table 1 that the simulation results show that *ψ*_*i*_ = 0 and therefore the best performing model is the Poisson (model 2) with the presence of the epidemic component having the least AIC = 1626.58. This implies that in the simulated data there was no overdispersion but rather temporal dependence.

**Table 1:** Simulation results including Parameter estimates, Standard errors and measure of model Goodness of Fit

Parameter	Model1	Model2	Model3	Model4
	(*ψ*=0 *λ* =0)	(*ψ*=0 *λ* ≠0)	(*ψ*≠0 *λ* =0)	(*ψ*≠0 *λ* ≠0)
*ψ*_1_	–	–	0.0000 (0.0000)	0.0000 (0.0000)
*ψ*_2_	–	–	0.0000 (0.0000)	0.0000 (0.0001)
*λ*_1_	–	0.1730 (0.3135)	–	0.1743 (0.3072)
*λ*_2_	–	0.4337 (0.2010)	–	0.4482 (0.2115)
*ϕ*_1_	0.4727 (0.2262)	0.4586 (0.2300)	0.4726 (0.3092)	0.4585 (0.2300)
*ϕ*_2_	0.8123 (0.0420)	0.0963 (0.2204)	0.3034 (0.2204)	0.1485 (0.2424)
*AIC*	1644.14	1626.58	1636.92	1630.33

### Application on data

Let {*y*_*it*_, *i* = 1, 2; *t* = 1,  … , 48} be the time series of virus counts for RSV (*y*_1*t*_) and HMPV (*y*_2*t*_) over the 48 months study time-frame. There were only two oscillations in a year for each of the two viruses to complete a cycle hence two harmonic waves (s = 2) were included in the model. The bivariate model for the two time series is therefore:

$$ \log \left(\begin{array}{c}{\mu}_{1,t}\\ {}{\mu}_{2,t}\end{array}\right)=\left(\begin{array}{c}{\lambda}_{1,t-1}\\ {}{\phi}_{2,t-1}\end{array}\begin{array}{c}{\phi}_{1,t-1}\\ {}{\lambda}_{2,t-1}\end{array}\right)\left(\begin{array}{c}{y}_{1,t-1}\\ {}{y}_{2,t-1}\end{array}\right)+\left(\begin{array}{c}{\tau}_{1,1}\ {\tau}_{1,2}\ {\tau}_{1,3}\ {\tau}_{1,4}\\ {}{\tau}_{2,1}\ {\tau}_{2,2}\ {\tau}_{2,3}\ {\tau}_{2,4}\end{array}\right)\left(\begin{array}{c}{x}_{1,t-1}\ \\ {}{x}_{2,t-1}\ \\ {}{x}_{3,t-1}\\ {}{x}_{4,t-1}\end{array}\right)+{\eta}_t\left(\begin{array}{c}{\nu}_{1,t}\\ {}{\nu}_{2,t}\end{array}\right), $$

where

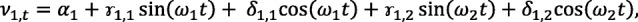

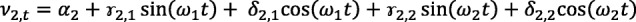





and *x*_1, *t* − 1_, *x*_2, *t* − 1_, *x*_3, *t* − 1_ and *x*_4, *t* − 1_ are the climatic factors representing rainfall, wind speed, mean dew point and visibility respectively. The term *η*_*t*_ corresponds to an offset term in the model (the monthly varying population counts at time *t*).

The models were compared for their fit to the epidemic data. Naturally, models are compared for their performance based on the ability to fit well on the data and their reliability in predicting future epidemic outbreaks. Fundamentally, in our model fitting to data we searched for the model that provided the best trade-off between the fit to data and the model structure complexity. Often, approaches such as the Akaike information criterion (AIC) and Bayesian information criterion (BIC) are sufficient for ranking and selecting the best performing models. However, when the data is non-Gaussian and the model is Bayesian, like in our case, then the deviance information criterion (DIC) is more appropriate. For the comparison of our models we used the DIC proposed by Spiegelhalter et al. [24], specifically for Bayesian-based models and it is a Bayesian generalization of the AIC and BIC. The model with the smallest DIC value gives the better trade-off between model fit and complexity; therefore, it is considered as the model that best predicts a replication of a data set with a similar structure as that which was observed currently [25].

To further assess the model performance with regards to the parameters, sensitivity analysis to alternative prior assumptions was performed because there are no true priors in the Bayesian analysis. In order to ensure reliable and robust results from our best model, it was crucial to verify how sensitive the resulting posteriors were for each prior input for the epidemic parameter *λ*_*it*_ and *ϕ*_*it*_, the parameter that quantifies the influence of one virus on the other. Therefore, we assumed independent gamma priors with uniform hyper-priors on the second parameter, *λ*_*it*_~*Ga*(*α*_*λ*,_*β*_*λ*_) and *β*_*λ*_~*Beta*(*a*, *b*) using *α*_*λ*_ = 1, *a* = 0.5 and *b* = 0.5. Similarly, for the influential parameter, we used the Beta distribution prior, *ϕ*_*it*_~*Beta*(*α*_*ϕ*,_*β*_*ϕ*_). To our understanding, this comparison of models has not yet been done using RSV and HMPV time series data. All the models in our work were run and tested in the statistical software WinBUGS version 14. The models differed on the epidemic part *ξ*_*i*, *t*_ by the assumptions made on the interactions between the viruses. We used 6 models depending on the assumptions applied as explained below with each model with a corresponding inclusion of climatic factors giving rise to a total of 12 models (Table 2). In model 1, it is assumed that the incidence rate is the same in every virus; hence, no interactions between the viruses. Model 2 assumes that there is the interaction between viruses where the sum of related viruses at the same time point has an equal rate. Models 3 and 4 are generalizations of models 1 and 2 respectively with a different rate for each virus. Models 5 and 6 generalize model 3 and 4 respectively with a different rate for each virus per time point.

**Table 2:** Models of the epidemic part *ξ*_*i*, *t*_ with assumptions made on interactions between the viruses with and without the climatic factors

Model	*ξ*_*i*, *t*_ (with climatic factors)	*ξ*_*i*, *t*_ (without climatic factors)
1	*λy*_*i*, *t* − 1_ + *τ*_*i*, *k*_*x*_*k*, *t* − 1_	*λy*_*i*, *t* − 1_
2	${\lambda y}_{i,t-1}+\phi \sum \limits_{j\ne i}{w}_{ji}{y}_{j,t-1}+{\tau}_{i,k}{x}_{k,t-1}$	${\lambda y}_{i,t-1}+\phi \sum \limits_{j\ne i}{w}_{ji}{y}_{j,t-1}$
3	*λ*_*i*_*y*_*i*, *t* − 1_ + *τ*_*i*, *k*_*x*_*k*, *t* − 1_	*λ*_*i*_ *y*_*i*, *t* − 1_
4	${\lambda}_i{y}_{i,t-1}+\sum \limits_{j\ne i}{w}_{ji}{\phi}_i{y}_{j,t-1}+{\tau}_{i,k}{x}_{k,t-1}$	${\lambda}_i{y}_{i,t-1}+\sum \limits_{j\ne i}{w}_{ji}{\phi}_i{y}_{j,t-1}$
5	*λ*_*i*, *t* − 1_*y*_*i*, *t* − 1_ + *τ*_*i*, *k*_*x*_*k*, *t* − 1_	*λ*_*i*, *t* − 1_ *y*_*i*, *t* − 1_
6	${\lambda}_{i,t-1}{y}_{i,t-1}+\sum \limits_{j\ne i}{w}_{ji}{\phi}_{i,t-1}{y}_{j,t-1}+{\tau}_{i,k}{x}_{k,t-1}$	${\lambda}_{i,t-1}{y}_{i,t-1}+\sum \limits_{j\ne i}{w}_{ji}{\phi}_{i,t-1}{y}_{j,t-1}$

The best model was then evaluated on whether; there were substantial interactions between cases of RSV and HMPV (alternatively stated as *ϕ*_RSV_ ≠ *ϕ*_HMPV_ ≠ 0), the existence of the influence of RSV on HMPV (*ϕ*_RSV_ = 0, *ϕ*_HMPV_ ≠ 0), the existence of the influence of HMPV on RSV (*ϕ*_HMPV_ = 0, *ϕ*_RSV_ ≠ 0) or there were no interactions at all (*ϕ*_RSV_ = *ϕ*_HMPV_ = 0).

## Results

**Fig. 1:**
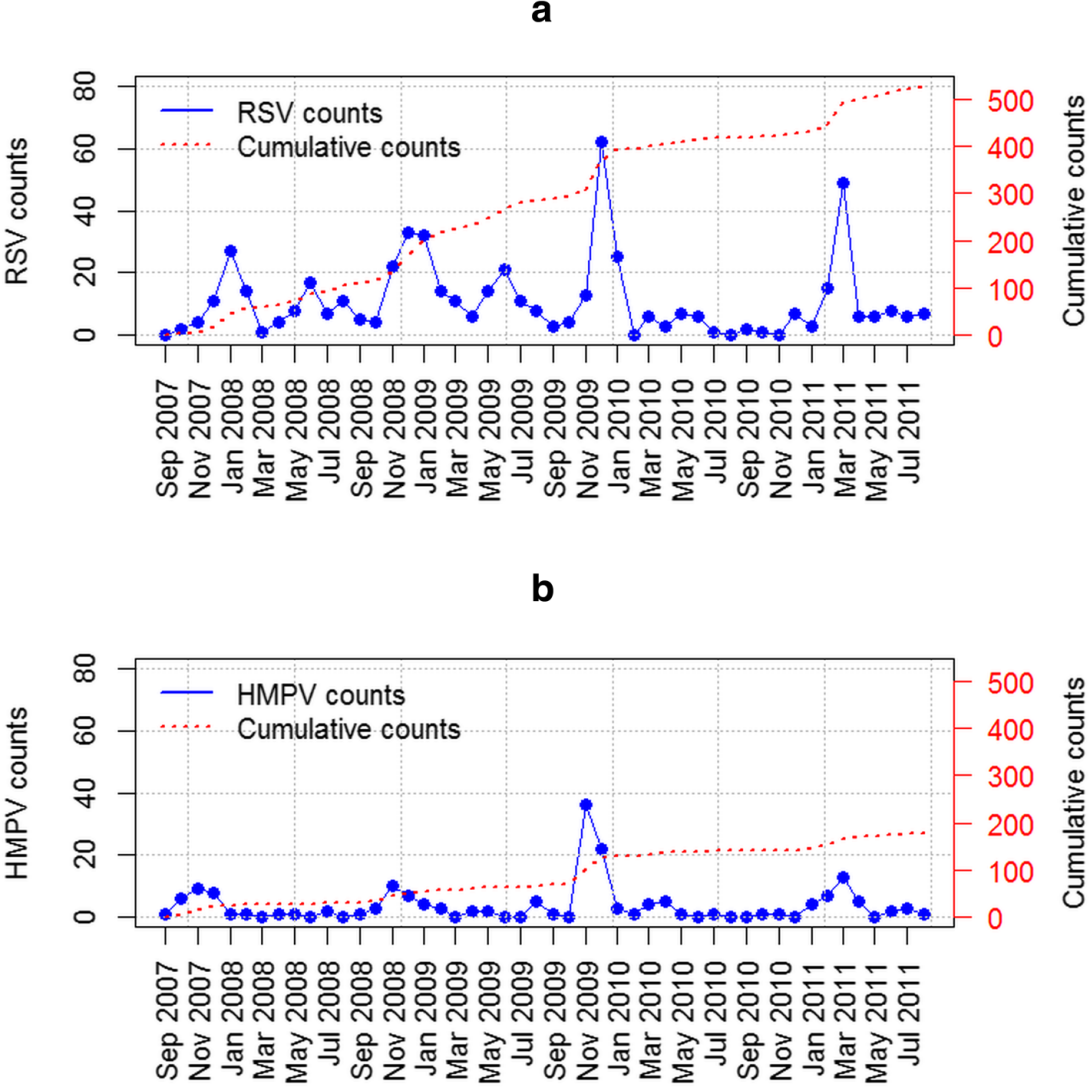
The monthly counts of epidemics (**a**) RSV and (**b**) HMPV plotted against time. The cumulative counts of HMPV cases were approximately 2.5 times less than the RSV counts for the same time-frame

**Fig. 2:**
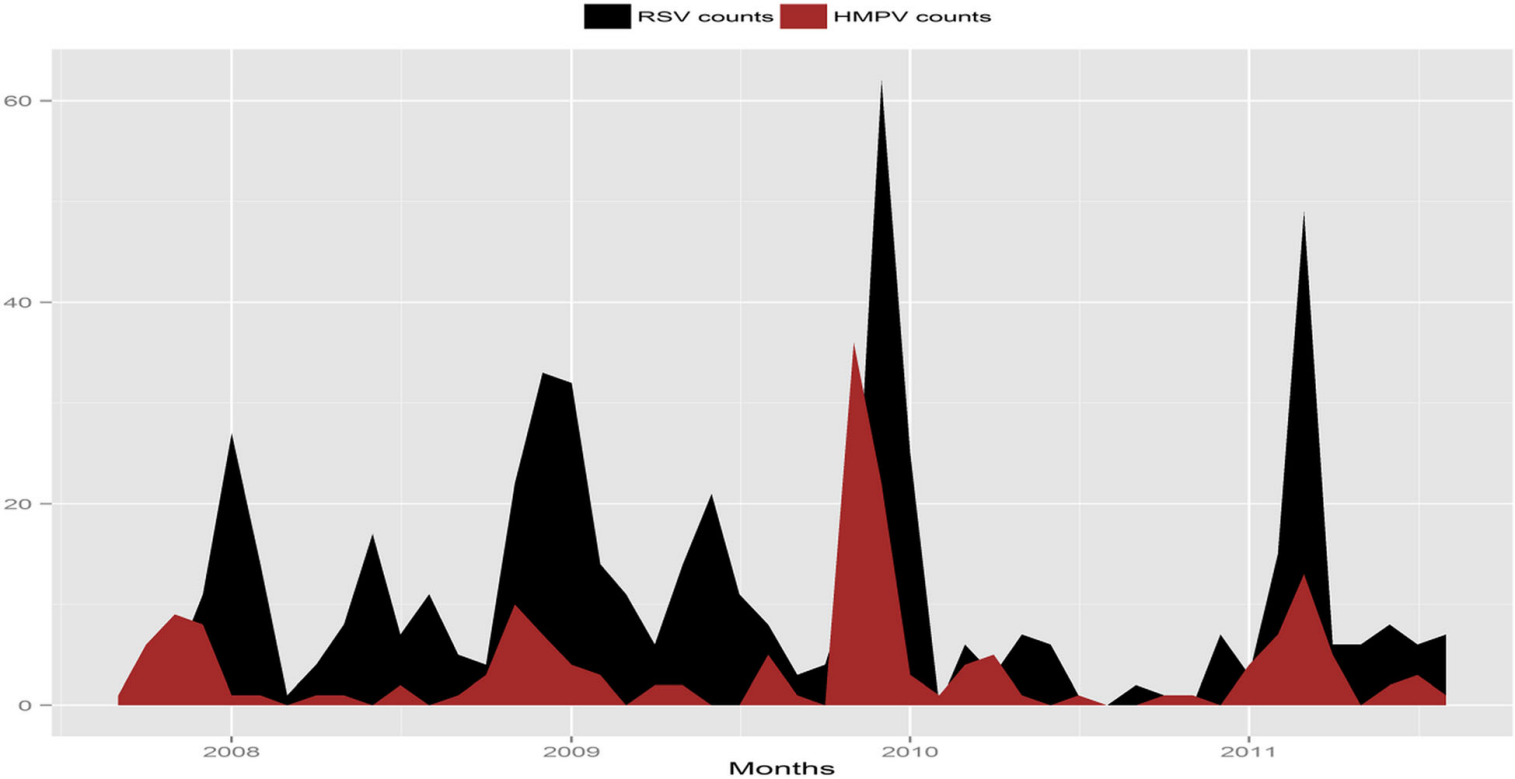
The monthly counts of RSV and HMPV plotted against time. Overall, the epidemics coincide in timing of their occurrence peaks, especially in March 2011

The monthly observed number of RSV and HMPV cases in Dadaab from September 2007 to August 2011 that were collected in the surveillance system was plotted (Fig. 1). The HMPV data shows a strong seasonality pattern as indicated by the four peaks during November of the years 2007, 2008 and 2009 while a fourth peak appears in March 2011 (Fig. 2). These HMPV peaks coincide with the RSV peaks.

We compared 12 models with various structures (Table 2) and the results for the DIC values are given in Table 3. Models 6 and 1 with climatic factors clearly out-perform the other models since, overall, they have lower DIC values. Model 6 with climatic factors had the least DIC value (173.52) and provided the best fit and explanation for the variation observed in the data. The models showed that the inclusion of climatic factors play an important role in the estimation of the number of cases for the two epidemics (RSV and HMPV). We further considered different scenarios on the best model with four sub-models (results are shown in Table 4).

**Table 3:** Comparison DIC values for different models

Model	1	2	3	4	5	6
DIC (with climatic factors)	490.43	558.30	559.46	558.45	502.17	173.52
DIC (without climatic factors)	549.82	541.11	548.44	536.09	571.72	744.22

**Table 4:** Four sub-models from the best model. The symbols “−” and “√” mean the absence and presence of interactions, respectively. Model 6 (i) no interactions between HMPV and RSV (*ϕ*_HMPV_ = *ϕ*_RSV_ = 0); Model 6 (ii) influence of HMPV on RSV (*ϕ*_RSV_ ≠ 0, *ϕ*_HMPV_ = 0), Model 6 (iii) influence of RSV on HMPV (*ϕ*_RSV_ = 0, *ϕ*_HMPV_ ≠ 0) and Model 6 (iv) interactions between HMPV and RSV (*ϕ*_HMPV_ ≠ *ϕ*_RSV_ ≠ 0)

Model	HMPV→RSV	RSV → HMPV	DIC
6(i)	–	–	543.68
6(ii)	√	–	457.61
6(iii)	–	√	112.14
6(iv)	√	√	173.52

**Fig. 3:**
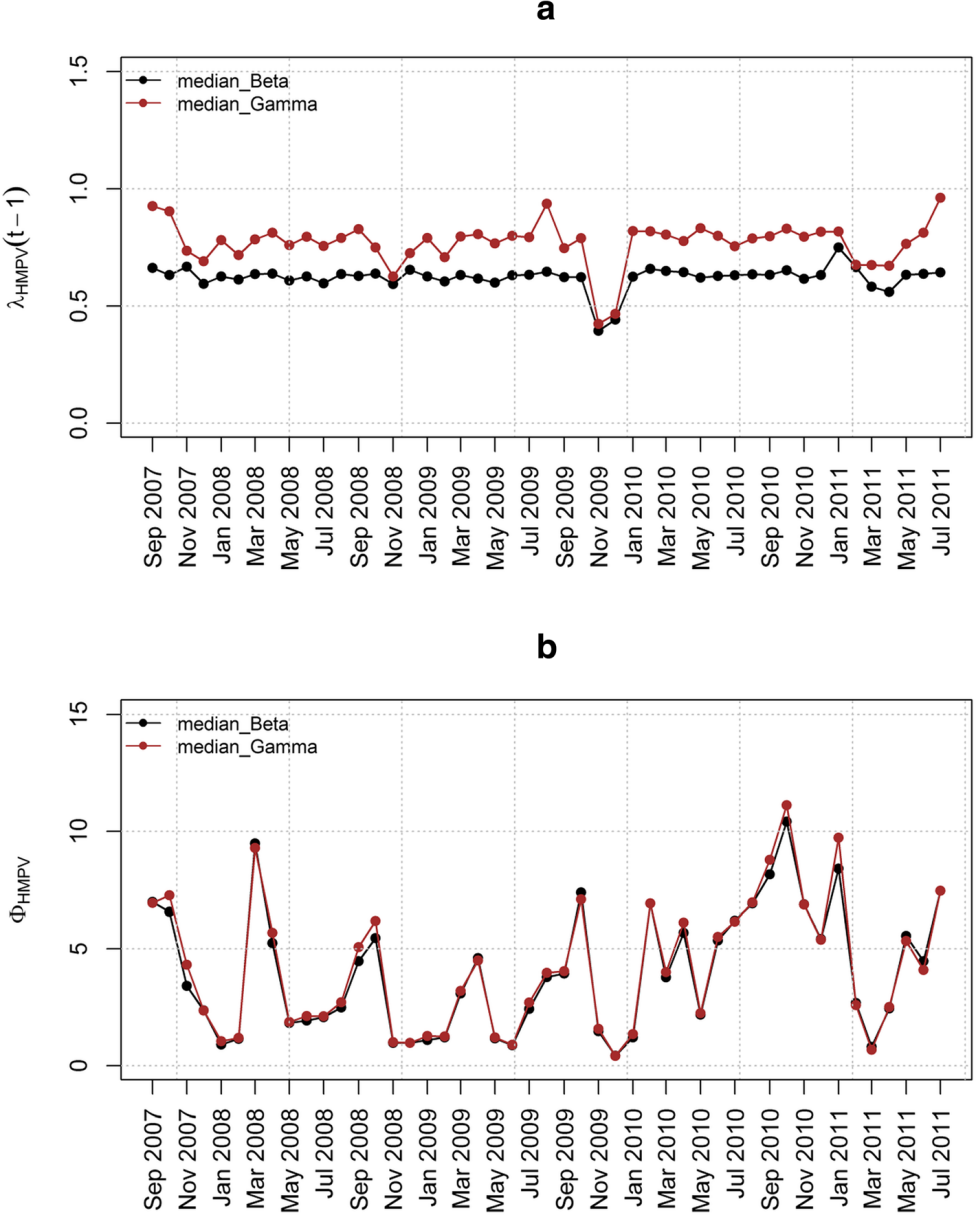
Posterior median and point-wise 95% credibility intervals for the best model. Plots showing the Posterior median and point-wise 95% credibility interval of (**a**) *λ*_HMPV_ and (**b**) *ϕ*_HMPV_ for model 6(iii)

Model 6(i) in Table 4 does not allow for interactions between HMPV and RSV (*ϕ*_HMPV_ = *ϕ*_RSV_ = 0) and its DIC value is 543.68. Model 6(ii) includes the influence of HMPV on RSV with the influence of RSV on HMPV equal to zero. This model yielded a DIC value of 457.61. Model 6(iii) includes the influence of RSV on HMPV where the influence of HMPV on RSV is zero. Compared to the others, this model yielded the smallest DIC value of 112.14 (Table 4). This implies that the two viruses can present as a co-infection where HMPV incidence is increased by increases in RSV. The results from sensitivity analysis shown in Fig. 3, indicates that this model is robust and insensitive to the prior distribution since its posterior distribution did not dramatically change upon altering the base prior parameter values. Model 6(iv) has both the influence of RSV on HMPV and the influence of HMPV on RSV which is the full model with a DIC value of 173.52 (Table 4). This indicates that the additional parameter (i.e., the influence of HMPV on RSV) into model 6(iii) does not significantly improve the model fit to data.

**Fig. 4:**
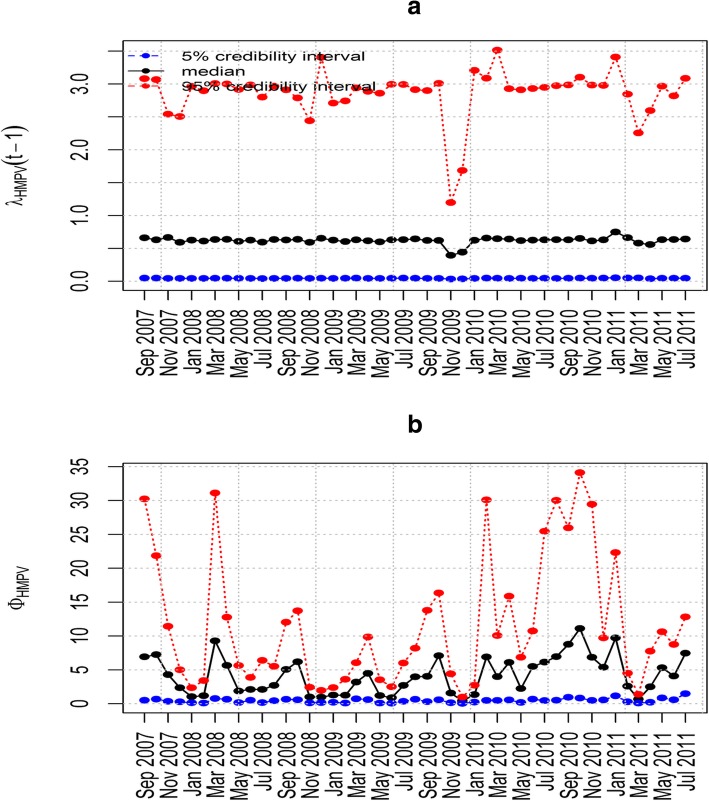
Posterior median values for the priors with Gamma and Beta distributions for the best model. Plots showing the Posterior median values of (**a**) *λ*_HMPV_ and (**b**) *ϕ*_HMPV_ for model 6(iii). Median_Beta and median_Gamma are the posterior medians from the Beta distribution and the Gamma distribution priors respectively

**Fig. 5:**
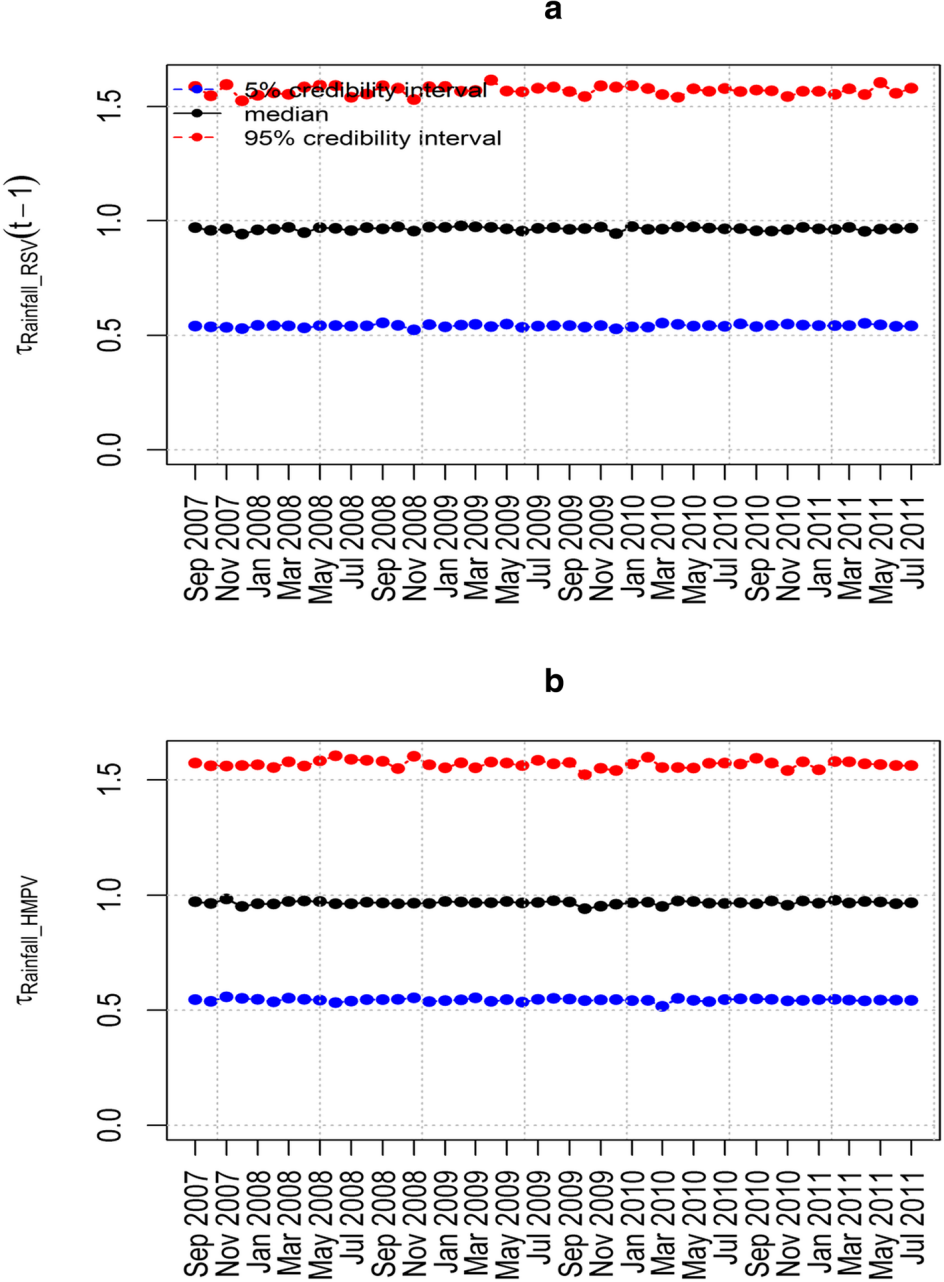
Posterior median and point-wise 95% credibility intervals for the best model. Plots showing the Posterior median and point-wise 95% credibility interval of (**a**) *τ*_Rainfall _ RSV_ and (**b**) *τ*_Rainfall _ HMPV_ for model 6(iii)

**Fig. 6:**
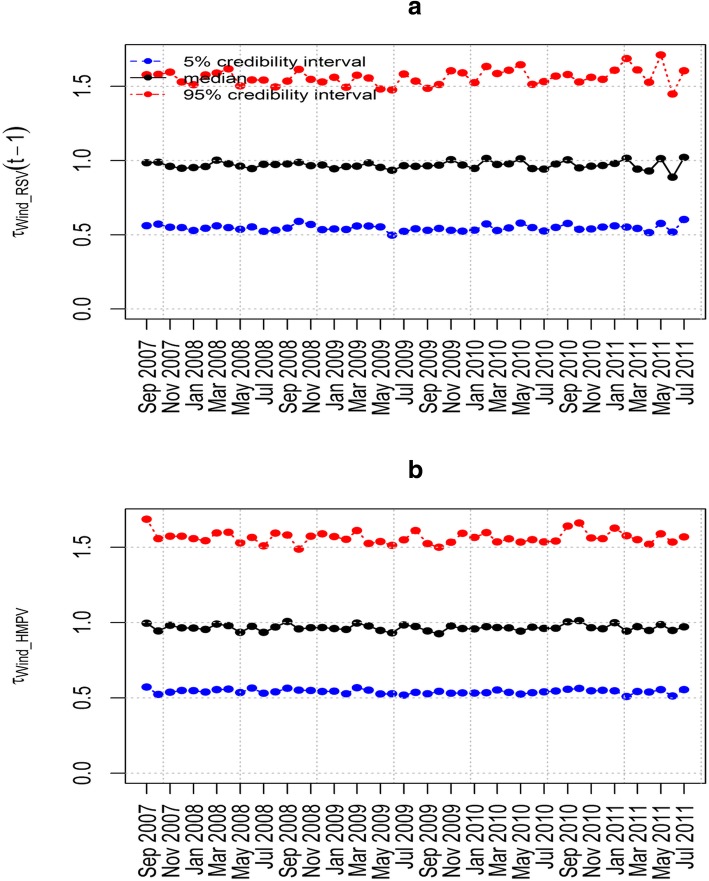
Posterior median and point-wise 95% credibility intervals for the best model. Plots showing the Posterior median and point-wise 95% credibility interval of (**a**) *τ*_Wind _ RSV_ and (**b**) *τ*_Wind _ HMPV_ for model 6(iii)

**Fig. 7:**
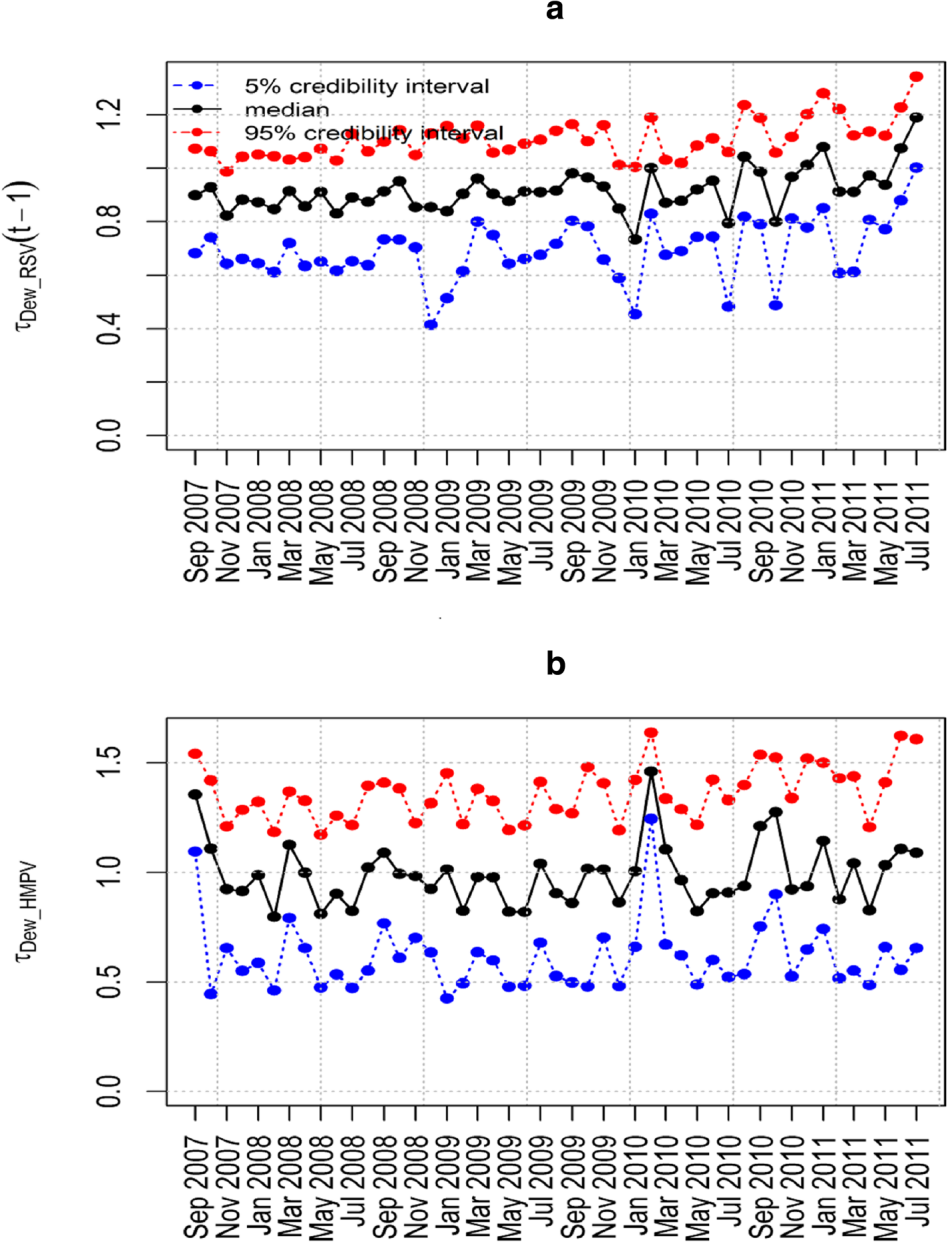
Posterior median and point-wise 95% credibility intervals for the best model. Plots showing the Posterior median and point-wise 95% credibility interval of (**a**) *τ*_Dew _ RSV_ and (**b**) *τ*_Dew _ HMPV_ for model 6(iii)

**Fig. 8:**
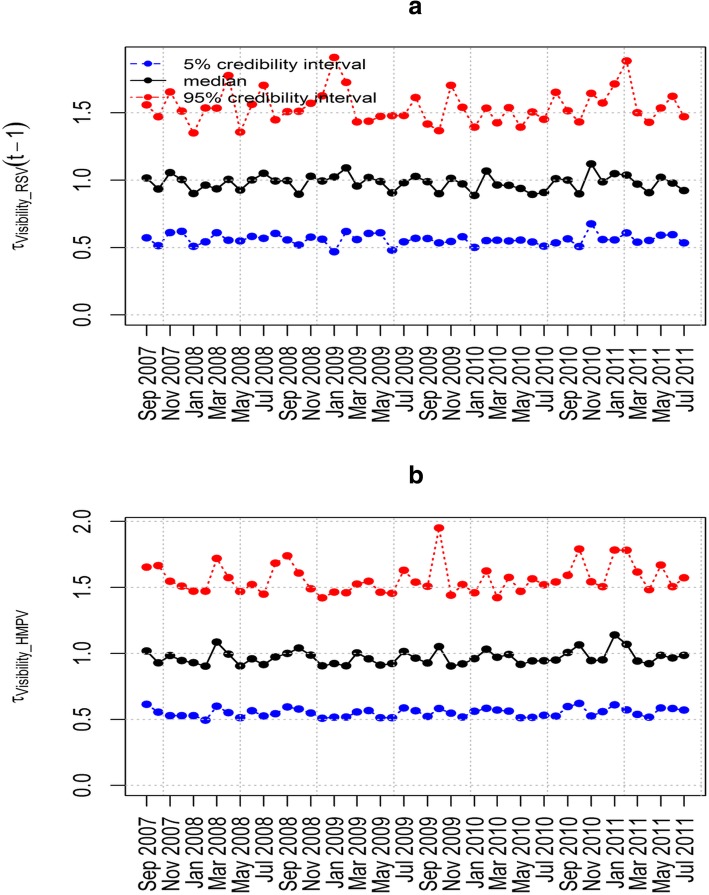
Posterior median and point-wise 95% credibility intervals for the best model. Plots showing the Posterior median and point-wise 95% credibility interval of (**a**) *τ*_Visibility _ RSV_ and (**b**) *τ*_Visibility _ HMPV_ for model 6(iii)

The epidemic parameter *λ*_HMPV_ for model 6(iii) in Fig. 4(a) does not exceed the value 1. This implies that the time series is stable without a detection of an outbreak of HMPV due to the influence of RSV. Figure 4(b) shows the influence of RSV on HMPV with biannual peaks noted over the study period. The other parameters estimated in this model are shown in Table 5 that includes the posterior median and point-wise 95% credibility intervals. In particular, from Table 5, the posterior median and the point-wise 95% credibility intervals for the over-dispersion parameters *ψ*_HMPV_ and ψ_RSV_ were 7.762(0.238, 116.1) and 4.688(0.090, 97.33) respectively. This indicates the existence of over-dispersion because the values for the parameters *ψ*_HMPV_ and ψ_RSV_ are greater than zero which relaxes our adoption of the negative-binomial modelling, despite in the simulation data there was over-dispersion detected. Figures 5, 6, 7 and 8 show the posterior median and point-wise 95% credibility intervals for the climatic factors.

**Table 5:** Posterior median and point-wise 95% credibility intervals for the best model

Parameter	5.0%	Median	95%
alpha1	−4.283	−3.998	−3.683
alpha2	−3.765	−3.765	− 3.481
delta11	−2.564	−2.564	−1.979
delta21	−4.783	−4.783	− 4.023
gamma11	−6.303	−5.653	−4.812
gamma21	−9.209	−7.965	−6.934
psi1	0.238	7.762	116.1
psi2	0.090	4.688	97.33

## Discussions

The RSV data shows bi-annual peaks of different severity during the rainy seasons in the Dadaab refugee camp (Kenya) [26, 27]. Wilkesmann et al. [28] showed that both HMPV and RSV cause similar symptoms and clinical severity with similar seasonality. A similar finding was reached by Kim et al. [29] who investigated the clinical and epidemiological assessment of HMPV and RSV in Seoul, Korea, 2003–2008. In their paper, Cuevas et al. [6] observed that HMPV incidence had increased with increases in RSV incidence. Another study in Yemen, children younger than 2 years identified co-infections of RSV and HMPV, and also showed that there were seasonal variations of RSV and HMPV with a peak of RSV in December and January and a peak of HMPV in February and March [30].

From our previous work using the same dataset, we noted a similar conclusion that the use of climatic factors explained the seasonality of RSV [27]. This implies that having considered the different rate for each virus at every time point, the models with the best fit to data were those with climatic factors. In our study, we have shown that the incidence of RSV influenced that of HMPV from the best model fit. It is therefore crucial to establish good RSV surveillance systems in developing countries to help understand the dynamics of the disease. This will aid in knowing when to put up an intervention to control for RSV and HMPV outbreaks. Some of the interventions include washing hands with soap and avoiding overcrowding. A similar observation was made by Lazar et al. who noted that HMPV did not contribute to the severity of RSV [31]. This is corroborated in findings from a similar investigation of the influence of RSV on HMPV by Greensill et al. [10] in which 70% of children infected with RSV were co-infected with HMPV. Elsewhere, Cuevas et al. [6] observed that HMPV incidence increased with increasing number of RSV cases suggesting the presence of a strong association between the dynamics of the two epidemics.

Some of the limitations of this study were that the available time series data for the viruses was only for a four-year time-frame which is short for time series analysis and that the climatic factors were from the neighboring weather station which is about 100 km away from the Dadaab camp. Nevertheless, the weather measurements are a good representation of the actual weather around Dadaab. There was no establishment of whether patients were co-infected during virus testing. We used the DIC which is an approximation of a penalized loss function based on the deviance to evaluate the models. The application is valid only when the number of parameters is much smaller than the number of independent observations [32]. The classical model selection was used that assumes that there is at least a best model for deducing inferences from the data. The criterion used to select the best model did not allow for the computation of weights of each fitted model to quantify for uncertainty, that is the model averaging techniques were not used [33].

## Conclusion

We provided a comprehensive comparison of RSV and HMPV in a refugee camp setting by using a bivariate non-Gaussian model to jointly model the epidemics. By comparing various model structures, we identified a model that satisfactorily fits the epidemic data, thereby explaining most of the observed variation therein. The models and estimated parameters also provided clues into the dynamics and stability of the two epidemics. Our results demonstrated the influence of RSV on HMPV while adjusting for climatic factors. The climatic factors played a significant role in explaining the influence of RSV incidence on HMPV incidence. These models are important to the public health implication since controlling the incidence of RSV would consequently reduce the incidence of HMPV.

## Data Availability

The data files and supplementary materials used for this study can be found at https://figshare.com/s/e8a735c22f554d8372e3 DOI: 10.6084/m9.figshare.5340724 .

## Abbreviations

AIC: Akaike information criterion
BIC: Bayesian information criterion
CDC: US Centers for Disease Control and Prevention
DIC: Deviance information criterion
HMPV: Human metapneumovirus
KEMRI: Kenya Medical Research Institute
LRTIs: Lower respiratory tract infections
RSV: Syncytial virus
RVIs: Respiratory virus infections
